# Clinical evidence for immune-based strategies in early-line multiple myeloma: current challenges in decision-making for subsequent therapy

**DOI:** 10.1038/s41408-023-00804-y

**Published:** 2023-03-22

**Authors:** Noopur Raje, María-Victoria Mateos, Shinsuke Iida, Donna Reece

**Affiliations:** 1 Department of Medical Oncology, Massachusetts General Hospital, Boston, MA USA; 2 University Hospital of Salamanca/IBSAL/Cancer Research Center-IBMCC (USAL-CSIC), Salamanca, Spain; 3 Department of Hematology and Oncology, Nagoya City University Graduate School of Medical Sciences, Nagoya, Japan; 4 Princess Margaret Cancer Centre, Toronto, ON Canada

**Keywords:** Haematological diseases, Myeloma

## Abstract

Almost all patients with multiple myeloma (MM) will eventually develop disease that has relapsed with or become refractory to available treatments and will require additional therapy. However, data are still lacking on how best to sequence regimens in the relapsed/refractory (RR) setting after the failure of early-line lenalidomide, bortezomib, and/or daratumumab, the most commonly used agents in clinical practice today. With the treatment landscape rapidly changing in response to emerging clinical trial data and approvals of several new drugs and additional combinations, it is critically important to focus on patients with RRMM. Variability in patient baseline characteristics, such as the number of prior lines of treatment, refractoriness to prior treatments, prior stem cell transplant, and timing and dosing of prior lenalidomide, makes it difficult to select the best options for patients with RRMM for whom first-line treatments have failed. The aim of this review is to provide both an overview of current therapies and future directions within the RRMM treatment landscape, and a framework for clinicians to choose the most promising next treatment option.

## Introduction

### Brief overview of multiple myeloma

Multiple myeloma (MM) is a cancer of the B-cell lineage resulting from the hyperproliferation of malignant plasma cells in the bone marrow, which is largely due to the dysregulation of oncogenic signaling pathways and abnormal immune function [1]. Most patients with MM experience relapse and eventually develop disease that is refractory to available treatments [1, 2]. Refractory disease can result from the presence of drug-resistant cells caused by multiple mechanisms, including mutations, reduced target expression, and changes in the tumor microenvironment [1–3]. Patient prognosis worsens with each relapse, and most high-risk patients, particularly elderly patients, will not receive a third line of therapy [4, 5]. Therefore, early-line treatments that provide disease control, delay relapse, prolong survival, are tolerable, and do not compromise the quality of life are critical.

The treatment landscape for MM continues to evolve, leading to improved outcomes. Data are still lacking, however, on how best to sequence regimens in relapsed/refractory MM (RRMM) [4, 6–8]. In this narrative review based on our expert opinion and an assessment of our clinical practice, we discuss the current treatment landscape for early-line treatment of MM, with a focus on immune-based agents, and associated clinical investigations.

### Current landscape of immune-based drugs in the early-line treatment of MM

The standard of care for MM includes combinations of drugs with different mechanisms of action, such as immunomodulatory drugs, monoclonal antibodies (mAbs), corticosteroids, proteasome inhibitors (PIs), and alkylating agents (Table 1). Almost all therapy combinations include a corticosteroid (dexamethasone or prednisone) and a PI (bortezomib, carfilzomib, or ixazomib), which induces apoptosis of malignant cells [2, 4, 6, 7, 9]. Immunomodulatory drugs currently recommended in MM are thalidomide, lenalidomide, and pomalidomide [2, 6, 7, 9]. Immunomodulatory drugs have a dual mechanism of action—direct tumor cell killing and enhancement of immune function [10, 11]. Specifically, they bind to cereblon, a component of the E3 ubiquitin ligase complex, leading to the degradation of the transcription factors Ikaros and Aiolos and resulting in the reactivation of apoptotic pathways in MM cells and enhancement of innate and adaptive immune cell function [10, 11]. mAbs targeting CD38 have also emerged as an important class of drugs in MM [12]. Daratumumab and isatuximab bind to CD38, a cell surface receptor highly expressed in myeloma cells and several types of immune cells, and exert their action through Fc-dependent mechanisms and immunomodulatory effects [3, 13]. Fc-dependent mechanisms involve antibody-dependent cellular cytotoxicity, antibody-dependent cellular phagocytosis, and complement-dependent cytotoxicity, which lead to lysis or phagocytosis of myeloma cells. The immunomodulatory effects of anti-CD38 mAbs promote T-cell proliferation and effector function through inhibition of CD38 enzymatic activity, which reduces adenosine immunosuppressive activity and elimination of CD38^+^ immunosuppressive cells [14]. mAbs targeting other myeloma cell epitopes have also been developed [15, 16]. Elotuzumab, a humanized IgG1 mAb targeting the SLAMF7 protein that is expressed on myeloma cells independent of cytogenetic abnormalities, mediates myeloma cell killing through mechanisms similar to those of the aforementioned anti-CD38 mAbs [15, 16]. Additional drugs considered within combination regimens are alkylating agents (e.g., cyclophosphamide), which cause DNA damage, and panobinostat, an inhibitor of the enzyme histone deacetylase, which activates the expression of tumor suppressor genes through the opening of chromatin structures initially silenced through histone acetylation [17]. Selinexor, an exportin-1 inhibitor, is an approved drug in MM that inhibits the nuclear export of tumor suppressor proteins and oncoproteins [18, 19]. Antibody-drug conjugates (ADCs) and chimeric antigen receptor (CAR) T-cell-directed therapies that target B-cell maturation antigen (BCMA), such as idecabtagene vicleucel (ide-cel) and ciltacabtagene autoleucel (cilta-cel), are also emerging as standard of care regimens in MM and are detailed later in this review, as well as cereblon E3 ligase modulators (CELMoD^®^ agents).

**Table 1:** Current standard of care regimens in multiple myeloma^a^.

	Corticosteroids	Immunomodulatory agents	Proteasome inhibitors	Anti-CD38 mAbs	Alkylating agents	Anti-SLAMF7 mAb	Nuclear export inhibitor	Bcl-2 inhibitor
**Currently available agents**	DEX PRED	THAL LEN POM	BORT CFZ IXA	DARA ISA	Cy MEL	ELO	SEL	VEN
**NDMM: ASCT eligible**	DEX	THAL	BORT	—	—	—	—	—
	DEX	—	BORT	—	Cy^b^	—	—	—
	DEX^c^	LEN^c^	BORT^c^	—	—	—	—	—
	DEX	THAL	BORT	DARA	—	—	—	—
	DEX	LEN^d^	BORT	DARA	—	—	—	—
	DEX	LEN	CFZ^d^	—	—	—	—	—
**NDMM: ASCT ineligible**	DEX^e^	LEN^e^	BORT^f^	—	—	—	—	—
	DEX^e^	LEN^e^	—	DARA^e^	—	—	—	—
	DEX (low-dose)^f^	LEN^f^	—	—	—	—	—	—
	PRED	—	BORT	DARA	MEL	—	—	—
	DEX^g^	—	BORT^g^	—	Cy^g^	—	—	—
**RRMM:**								
**With prior lenalidomide**^**h**^	DEX	POM	BORT or CFZ or IXA	—	—	—	—	—
	DEX	—	BORT or CFZ	DARA	—	—	—	—
	DEX	—	CFZ	ISA	—	—	—	—
	DEX	—	CFZ	—	Cy	—	—	—
	DEX	POM	—	—	—	ELO	—	—
	DEX	POM	—	DARA or ISA	—	—	—	—
	DEX	—	BORT	—	—	—	SEL	—
	DEX	—	BORT	—	—	—	—	VEN^i^
**With prior daratumumab**^**h**^	DEX	POM^j^	BORT	—	—	—	—	—
	DEX	POM^j^	—	ISA	—	—	—	—
	DEX	—	CFZ	ISA	—	—	—	—
	DEX	LEN	—	—	—	ELO	—	—
	DEX	—	BORT	—	—	—	SEL	—
	DEX	—	BORT	—	—	—	—	VEN^i^
	DEX	POM	—	—	—	ELO	—	—

— agent not applicable for given combination regimen.

*ASCT* autologous stem cell transplant, *BORT* bortezomib, *CFZ* carfilzomib, *Cy* cyclophosphamide, *DARA* daratumumab, *DEX* dexamethasone, *ELO* elotuzumab, *ISA* isatuximab, *IXA* ixazomib, *LEN* lenalidomide, *MEL* melphalan, *NDMM* newly diagnosed multiple myeloma, *POM* pomalidomide, *PRED* prednisone, *RRMM* relapsed/refractory multiple myeloma, *SEL* selinexor, *THAL* thalidomide, *VEN* venetoclax.

^a^Each row represents a combination regimen recommended for the disease state given in the first column.

^b^May be substituted for an immunomodulatory drug if not available in select countries.

^c^May be preferred based on risk-benefit profile.

^d^Studies still ongoing on this combination.

^e^Use for fit patients.

^f^Use for unfit patients until disease progression.

^g^Additional lenalidomide-free regimen recommended in the United States.

^h^Evidence supporting the efficacy of these regimens is limited in these populations.

^i^Patients with t(11;14).

^j^Use for patients with prior lenalidomide exposure.

For patients with newly diagnosed MM (NDMM), immunomodulatory drugs combined with PIs and a steroid are widely used, and more recently, daratumumab-based combinations have been recommended [6, 9]. Initial therapy can vary across countries depending on drug availability and patient eligibility for autologous stem cell transplant (ASCT). For those who are transplant eligible (TE), the most common standard treatment is lenalidomide, thalidomide, or cyclophosphamide added to a bortezomib-dexamethasone backbone as induction therapy prior to ASCT, followed by continuous lenalidomide maintenance therapy until disease progression (Table 1) [6, 9, 20]. Chemotherapy with high-dose melphalan (200 mg/m^2^ intravenous) is the standard conditioning regimen before ASCT [6, 9]. Among these combinations, lenalidomide-bortezomib-dexamethasone has been suggested to offer the best risk-benefit profile [9]. If an immunomodulatory drug is not available in certain countries, cyclophosphamide may be substituted [6, 9]. The inclusion of daratumumab or isatuximab as an early-line option is changing the treatment landscape, owing to the approval of combination regimens such as daratumumab-bortezomib-thalidomide-dexamethasone in TE patients, daratumumab- or isatuximab-carfilzomib-dexamethasone in patients with RRMM who have received 1 to 3 prior lines of therapy; and daratumumab-bortezomib-melphalan-prednisone or daratumumab-lenalidomide-dexamethasone in those who are transplant ineligible (TI) [2, 21–23]. The phase 3 CASSIOPEIA study demonstrated that the addition of daratumumab to thalidomide-bortezomib-dexamethasone increased the depth of response and improved rates of progression-free survival (PFS) in patients with TE NDMM [24]. The addition of daratumumab to lenalidomide-bortezomib-dexamethasone regimens also improved the depth of response in patients with TE NDMM in the phase 2 GRIFFIN study, a finding that is being evaluated further in the phase 3 PERSEUS study [21, 25, 26]. Daratumumab was approved by the US Food and Drug Administration (FDA), European Commission (EC), and Health Canada in 2019 in combination with thalidomide-bortezomib-dexamethasone; [21–23] to date, studies are ongoing for the combination with lenalidomide-bortezomib-dexamethasone.

In TI patients, standard treatments include lenalidomide-bortezomib-dexamethasone or lenalidomide-daratumumab-dexamethasone for fit patients and lenalidomide–low-dose dexamethasone for unfit patients (Table 1) [2, 6, 9, 27, 28]. The effectiveness of these regimens may partially depend on the characteristics of the patient population. For example, compared with lenalidomide-dexamethasone, lenalidomide-bortezomib-dexamethasone resulted in significant improvements in PFS and OS only in patients aged <65 and <75 years, respectively, in phase 3 SWOG S0777 trial [29]. In the phase 3 MAIA trial, the PFS benefit with daratumumab-lenalidomide-dexamethasone vs lenalidomide-dexamethasone was maintained in the subgroup of patients aged >75 years (median PFS, not reached [NR] vs 31.9 months, respectively), although inferential statistical testing was not performed for these data [30]. The bortezomib dosing frequency can also be modified without compromising the efficacy of this regimen, as evidenced by the robust PFS benefit observed in a phase 2 study of lenalidomide, bortezomib and dexamethasone (RVd) lite (administered over a 35-day cycle: oral [PO] lenalidomide 15 mg on days 1–21; subcutaneous bortezomib 1.3 mg/m^2^ on days 1, 8, 15, and 22; and PO dexamethasone 20 mg on days 1, 2, 8, 9, 15, 16, 22, and 23) in patients with TI NDMM (median PFS, 41.9 months) [31]. Lenalidomide is an important component of these regimens, as it has been shown to delay initiation of second-line therapy for >3 years [27]. Daratumumab is also recommended for use without lenalidomide when added to bortezomib-melphalan-prednisone [6, 9, 21, 22]. The inclusion of daratumumab within these combinations was based on results from the phase 3 MAIA and ALCYONE studies, which demonstrated longer PFS when combined with lenalidomide-dexamethasone or bortezomib-melphalan-prednisone, respectively [30, 32]. The authors consider the current standard of care in this setting to be daratumumab-lenalidomide-dexamethasone, based on more recently reported survival data from the MAIA trial, which showed longer PFS (daratumumab-lenalidomide-dexamethasone vs lenalidomide-dexamethasone: NR vs 34.4 months) in patients with TI NDMM [33]. An additional lenalidomide-free recommended regimen in the United States is cyclophosphamide-bortezomib-dexamethasone [6].

As lenalidomide-based therapies have become common in frontline therapy in NDMM, pomalidomide- and daratumumab-based regimens as next-line options have been studied in recent clinical trials. The following triplet combinations are currently recommended based on ASCO/CCO, IMWG, and EHA-ESMO guidelines for patients with RRMM previously exposed to lenalidomide (Table 1): pomalidomide-dexamethasone plus a PI (bortezomib, ixazomib, or carfilzomib), an anti-CD38 mAb (daratumumab or isatuximab), or an anti-SLAMF7 mAb (elotuzumab); daratumumab-dexamethasone plus a PI (bortezomib or carfilzomib); and isatuximab-carfilzomib-dexamethasone [6, 7, 9]. The approval of daratumumab-based combinations in the frontline setting has introduced additional complexity in the selection of next-line options [9]. For patients previously exposed to daratumumab, next-line options may include PIs and immunomodulatory agents, particularly pomalidomide-bortezomib-dexamethasone for patients previously also exposed to lenalidomide [6, 7, 9]. Isatuximab, which received approval from the FDA, EC, and Health Canada in 2020 for use in combination with pomalidomide-dexamethasone and carfilzomib-dexamethasone [34, 35], may be an option since the epitopes of daratumumab and isatuximab do not overlap, and they induce different structural changes within the CD38 protein that may lead to differential tumor cell killing; evidence-based data, however, are lacking in this regard [7, 36]. In a phase 2 study of isatuximab monotherapy in patients with RRMM and daratumumab-refractory disease, the primary endpoint of overall response rate (ORR) was not met; the disease control rate was 37.5% but was greater in patients with daratumumab washout periods of ≥6 months vs <3 months (58.3 vs 28.6%, respectively) [37]. Further study will be necessary to determine the potential of isatuximab monotherapy or combination therapy for patients who have developed the daratumumab-refractory disease. Additionally, based on its approval by the FDA and EC, elotuzumab may be used in combination with pomalidomide-dexamethasone or lenalidomide-dexamethasone [2, 9, 15, 38].

An alternative to pomalidomide or daratumumab is switching from bortezomib to carfilzomib within a triplet-combination that also includes cyclophosphamide, as PI sensitivity is often retained following bortezomib exposure. Carfilzomib has been shown to induce apoptosis in bortezomib-resistant MM cell lines and in patient samples [39]. However, the clinical benefit of treatment with carfilzomib or bortezomib may depend on the patient population. In the phase 3 ENDURANCE trial of carfilzomib vs bortezomib in combination with lenalidomide-dexamethasone in patients with NDMM, the median PFS was similar between groups (34.6 vs 34.4 months, respectively) [40]. However, the composite rates of grade ≥3 cardiac, pulmonary, and renal toxicities were greater with carfilzomib-lenalidomide-dexamethasone than with bortezomib-lenalidomide-dexamethasone (16 vs 5%, respectively), whereas the rates of grade ≥3 peripheral neuropathy were lower (1% vs 8%). Consequently, the toxicologic profile associated with carfilzomib may limit the use of this agent in patients with underlying cardiopulmonary or renal comorbidities, and the peripheral neuropathy associated with bortezomib may limit its use in patients with neurological comorbidities. However, carfilzomib has also shown a survival benefit in patients with RRMM, as evidenced in the phase 3 ENDEAVOR trial, in which patients who had received 1 to 3 prior lines of treatment had an increased median overall survival (OS) with carfilzomib-dexamethasone vs bortezomib-dexamethasone (47.8 vs 38.8 months, respectively; hazard ratio, 0.76; 95% CI, 0.63–0.92) [41]. Furthermore, cyclophosphamide added to carfilzomib-dexamethasone has demonstrated clinical benefit in patients with the lenalidomide-refractory disease [42]. Another option in this setting is the combination of selinexor with bortezomib-dexamethasone, as evaluated in the phase 3 BOSTON trial [43]. Pomalidomide has also been shown to inhibit the proliferation of lenalidomide-resistant MM cell lines [16]. This observed in vitro efficacy is supported by the results of the phase 3 OPTIMISMM trial, which found that treatment with pomalidomide, bortezomib, and low-dose dexamethasone improved PFS compared with bortezomib and low-dose dexamethasone in patients with RRMM and lenalidomide-refractory disease (median PFS, 9.5 vs 5.6 months, respectively) [44, 45]. Immunophenotypic profiling of peripheral blood samples from patients treated with daratumumab, pomalidomide, and low-dose dexamethasone in arm B of phase 2 MM-014 trial further supported the efficacy of pomalidomide in patients with the lenalidomide-refractory disease [46]. Pomalidomide mediated increases in proliferating T cells, increases in HLA-DR^+^ activated T cells, and expansion in the effector memory T-cell compartment in patients with the lenalidomide-refractory disease and the total population, suggesting that the efficacy of pomalidomide is maintained in patients with the lenalidomide-refractory disease [46]. A more detailed review of the treatment of patients with lenalidomide exposure, including lenalidomide-refractory disease, can be found in Moreau et al. [8] and the International Myeloma Working Group guidelines [7].

### Current investigations in RRMM and associated data gaps

The phase 3 FIRST, SWOG, Myeloma XI, and CALGB studies collectively established the role of frontline lenalidomide until disease progression for patients with TE or TI NDMM [28, 29, 47, 48]. Additionally, with daratumumab being increasingly prescribed in the frontline setting due to its recent approval in many countries [21–23], it has become critical to study regimens that can be given to patients with MM refractory to lenalidomide or daratumumab early in their disease course, especially at first relapse. Despite the multitude of therapeutic options available for patients with RRMM, evidence of effectiveness in early lines of therapy and in patients who experienced lenalidomide treatment failure is limited [7, 8, 29]. This is, in part, because many of the recent phase 3 trials were designed prior to frontline lenalidomide becoming a frequently used treatment strategy. Additionally, evidence of treatment efficacy following the failure of early-line daratumumab is limited by the relatively low clinical trial enrollment of patients with daratumumab-refractory or -relapsed disease. Currently, pomalidomide-, carfilzomib- and anti-CD38–based regimens are options considered for next-line therapy after either lenalidomide or daratumumab and will be the focus of this review.

Table 2 provides a summary of patient baseline characteristics in phase 2 and 3 clinical trials of immune-based therapy in early-line RRMM that are focused on frontline exposure to lenalidomide. Some data regarding prior treatment with daratumumab in these patient populations are provided, although limited conclusions can be drawn about these subgroups due to small sample sizes. The following therapy combinations with the associated trials are included in this table: (a) pomalidomide-based regimens—pomalidomide-dexamethasone plus bortezomib (phase 3 OPTIMISMM), carfilzomib (phase 2 EMN011), or cyclophosphamide (phase 2 IC 2013-05); (b) anti-CD38 antibody-based regimens—daratumumab-dexamethasone plus pomalidomide (phase 3 APOLLO and phase 2 MM-014), bortezomib (phase 3 CASTOR), or carfilzomib (phase 3 CANDOR); and (c) isatuximab-dexamethasone plus pomalidomide (phase 3 ICARIA-MM) or carfilzomib (phase 3 IKEMA). Table 3 provides an overview of efficacy data from select phase 2 and 3 RRMM trials.

**Table 2:** Baseline characteristics relevant to lenalidomide and daratumumab refractoriness in phase 2 and 3 RRMM clinical trials.

	OPTIMISMM [45]	EMN011 [55, 94]	IC 2013-05 [61]	APOLLO [95]	MM-014 Arm B [58]	CASTOR [50]	CANDOR [96]	ICARIA-MM [59]	IKEMA [51–53]
**Triplet regimen studied**	POM/DEX + BORT	POM/DEX + CFZ	POM/DEX + Cy	DARA + POM/DEX	DARA + POM/DEX	DARA + BORT/DEX	DARA + CFZ/DEX	ISA + POM/DEX	ISA + CFZ/DEX
**Phase**	3	2	2	3	2	3	3	3	3
**Clinical trial identifier**	NCT01734928	NTR5349	NCT02244125	NCT03180736	NCT01946477	NCT02136134	NCT03158688	NCT02990338	NCT03275285
**Patients, n**	281	60	100	151	112	498	466	154	302
**Prior lines of therapy, median (range), n**	2 (1–2)	–	1	2 (1–5)	1 (1–2)	2 (1–10)	2 (1–2)	3 (2–4)	(1–≥3)
**Exposed to prior LEN, %**	100	100	100	100	100 (last line)	76 (IMiD)	42	100	78 (IMiD)
**Refractory to LEN, %** **Refractory to LEN** + **PI, %**	71 –	– –	0 –	79 42	75 –	32.9 (IMiD) –	33 –	94 72 (LEN in last line: 60)	– –
**Refractory to LEN-based maintenance, %**	–	95	–	–	–	–	–	–	–
**Exposed to prior daratumumab, %**	–	–	–	–	0	–	<1% (anti-CD38 antibody)	*n* = 1	–
**Refractory to daratumumab, %**	–	–	–	0	0	0	–	–	0
**Exposed to prior PI, %**	75	–	–	100	79.5	–	–	100	90
**BORT**	72	–	100	–	78	65.5	90	–	–
**CFZ**	3	–	–	–	10	–	–	–	–
**IXA**	3	–	–	–	4	–	–	–	–
**Refractory to PI, %**	13	–	–	47	–	–	–	77	–
**BORT**	9	100	–	–	–	0	29	–	–
**CFZ**	–			–		–	–	–	0
**Prior ASCT, %**	57	67	50	60	70	61	–	54	–
**Prior-treatment subgroup analyses**	LEN-refr; 1 prior therapy; LEN-refr + 1 prior therapy	–	–	LEN-refr	Prior line of therapy (1, 2); dose of prior LEN (≤10 mg, >10 mg); LEN-rel, LEN-refr; prior LEN + PI; ASCT	Prior line of therapy (1, 2, 3); prior BORT	Prior line of therapy (1, ≥2); prior PI; prior LEN exposure; refr to LEN; refr to BORT or IXA; prior immunomodulatory drug exp or refr	Prior line of therapy (2–3; >3); LEN-refr; PI-ref; LEN + PI refr; LEN (last line)-refr; PI (last line)-refr; prior ASCT	Prior line of therapy

–, not reported.^a^

*ASCT* autologous stem cell transplant, *BORT* bortezomib, *CFZ* carfilzomib, *Cy* cyclophosphamide, *DARA* daratumumab, *DEX* dexamethasone, *Exp* experienced, *IMiD* immunomodulatory drug, *ISA* isatuximab, *IXA* ixazomib, *LEN* lenalidomide, *PI* proteasome inhibitor, *POM* pomalidomide, *refr* refractory, *rel* relapsed, *RRMM* relapsed/refractory multiple myeloma.

^a^Unreported data could be due to the exclusion of patients with prior daratumumab exposure, including daratumumab-refractory disease or a lack of subgroup reporting in the respective trials.

**Table 3:** Summary of efficacy results of select phase 2 and 3 RRMM clinical trials^a^.

Trial^b^	OPTIMISMM *N* = 559 [45]	EMN011 *N* = 60 [55, 57, 94]	IC 2013-05 *N* = 100 [61]	APOLLO *N* = 304 [49, 97]	MM-014 *N* = 112 [58, 60]	CASTOR *N* = 498 [50, 54, 98]	CANDOR *N* = 466 [63, 96]	ICARIA-MM *N* = 307 [59, 99]	IKEMA *N* = 302 [52, 53, 100]
**Drug regimen**	POM/DEX + BORT *n* = 281	POM/DEX + CFZ *n* = 60	POM/DEX + Cy *n* = 97	DARA + POM/DEX *n* = 151	DARA + POM/DEX *n* = 112	DARA + BORT/DEX *n* = 251	DARA + CFZ/DEX *n* = 312	ISA + POM/DEX *n* = 154	ISA + CFZ/DEX *n* = 179
**Comparator**	DEX + BORT *n* = 278	NA	NA	POM + DEX *n* = 153	NA	BORT + DEX *n* = 247	CFZ + DEX *n* = 154	POM + DEX *n* = 153	CFZ + DEX *n* = 123
**ORR, n/N (%)**^**c,d**^	231/281 (82.2) vs 139/278 (50)	52/60 (86.7)	82/97 (84.5)	104/151 (69) vs 71/153 (46)	87/112 (77.7)	203/240 (84.6) vs 148/234 (63.2)	263/312 (84.3) vs 112/154 (72.7)	93/154 (60.4) vs 54/153 (35.3)	155/179 (86.6) vs 103/123 (83.7)
**CR rate, n/N (%)**^**d,e**^	44/281 (15.7) vs 11/278 (4.0)	19/60 (31.7)	1/97 (1.0)	37/151 (25) vs 6/153 (4)	30/112 (26.8)	72/240 (30.0) vs 23/234 (9.8)	103/312 (33.0) vs 20/154 (13.0)	7/154 (4.5) vs 3/153 (2.0)	79/179 (44.1) vs 35/123 (28.5)
**DCR, n/N (%)**^**d,f**^	263/281 (93.6) vs 245/278 (88.1)	—	85/97 (87.6)	141/151 (93.4) vs 135/153 (88.2)	105/112 (93.8)	—	—	136/154 (88.3) vs 116/153 (75.8)	—
**PFS follow-up, median, months**^**d**^	15.9	40	33.6	16.9	28.4	40.0	27.8 vs 27.0	11.6	44
**PFS, median (95% CI), months**^**d**^	11.2 (9.7–13.7) vs 7.1 (5.9–8.5)	26	34.2 (27.6-NE)	12.4 (8.3–19.3) vs 6.9 (5.5–9.3)	30.8 (19.3–NE)	16.7 vs 7.1	28.6 (22.7-NE) vs 15.2 (11.1–19.9)	11.5 (8.9–13.9) vs 6.5 (4.5–8.3)	35.7 (25.8–44.0) vs 19.2 (15.8–25.0)
**PFS in patients with prior LEN exposure, median (95% CI), months**^**d**^	9.5 (8.1–11.3)^g^ vs 5.6 (4.4–7.0)	—	—	9.9 (6.5–13.1)^g^ vs 6.5 (4.7–8.9)	23.7 (14.0–NE)^g^ NR (18.2–NE)^h^	7.8 vs 4.9	28.1 (20.3–NE)^g^ vs 11.1 (6.5–13.2)	—	—
**PFS in patients with prior LEN exposure, HR (95% CI)**^**d**^	0.65 (0.50–0.84)^g^	—	—	0.66 (0.49–0.90)^g^	—	0.44 (0.28–0.68)^g^	0.46 (0.28–0.73)^g^	0.59 (0.43–0.82)^g^	—
**OS follow-up, median, months**^**d**^	—	—	33.6	39.6	—	72.6	17.2 vs 17.1	35.3	—
**OS, median (95% CI), months** ^**d**^	—	—	NE (42.2–NE)	34.4 (23.7–40.3) vs 23.7 (19.6–29.4)	—	49.6 (42.2–62.3) vs 38.5 (31.2–46.2)	NR vs NR	24.6 (20.3–31.3) vs 17.7 (14.4–26.2)	—

— not reported,

*BORT* bortezomib, *CFZ* carfilzomib, *CI* confidence interval, *CR* complete response, *Cy* cyclophosphamide, *DARA* daratumumab, *DCR* disease control rate, *DEX* dexamethasone, *ISA* isatuximab, *NA* not applicable, *NE* not estimable, *NR* not reached, *ORR* overall response rate, *OS* overall survival, *POM* pomalidomide, *PFS* progression-free survival, *RRMM* relapsed/refractory multiple myeloma.

^a^Comparisons between trials are not recommended due to differences in clinical trial design, patient characteristics, and clinical practice between studies.

^b^Different citations represent different data cutoffs; the most recent data for response or survival outcomes from these sources are reported here.

^c^Overall response rate was defined as the proportion of patients who achieved a partial or complete response.

^d^Data were presented as drug regimen vs comparator.

^e^Defined as the proportion of patients who achieved a complete response or stringent complete response.

^f^Disease control rate was defined as the proportion of patients who achieved a complete response, partial response, minimal response, or stable disease.

^g^Patients with lenalidomide-refractory disease.

^h^Patients who experienced disease relapse following treatment with lenalidomide.

As shown in Table 2, the number of prior lines of therapy varied within individual trials and the pooling of these data creates difficulty in determining outcomes in early-line settings. OPTIMISMM, MM-014, and CANDOR trials evaluated patients with earlier lines of therapy and smaller ranges (1–2 prior lines for MM-014 and 1–3 for OPTIMISMM and CANDOR), while the ranges in CASTOR and APOLLO were higher (1–10 and 1–5, respectively). Despite these ranges, most trials included subanalyses to evaluate patients who received only 1 prior line of therapy; however, overall patient numbers were low.

Inclusion criteria for patients who have lenalidomide- or daratumumab-exposed or refractory disease were not commonly included in the design of earlier trials but are gaining more attention. The OPTIMISMM study was the first phase 3 trial designed to specifically include patients previously exposed to lenalidomide, with the majority (71%) having lenalidomide-refractory disease [45]. In this trial, the median PFS with pomalidomide-bortezomib-dexamethasone was 9.5 months in patients with lenalidomide-refractory disease. In the APOLLO trial, all patients had previously received an immunomodulatory agent plus a PI, and 79% were refractory to lenalidomide; the median PFS with daratumumab-pomalidomide-dexamethasone was 9.9 months in patients with the lenalidomide-refractory disease [49]. In CASTOR and IKEMA, 76 and 78% of patients received a prior immunomodulatory agent, respectively, and 33% in CASTOR were considered to be lenalidomiderefractory [50–53]. In post hoc analyses of CASTOR (median follow-up, 40.0 months), the median PFS was 16.7 months in patients treated with daratumumab-bortezomib-dexamethasone, with a longer PFS noted in patients with 1 prior line of treatment [54]. The EMN011 trial was designed to select patients with refractory disease or first progression after having received lenalidomide maintenance therapy until progression as part of the EMN02 trial [55, 56]. In this trial (median follow-up, 40 months), patients treated with carfilzomib-pomalidomide-dexamethasone had a median PFS of 26 months from the date of registration [57]. All patients in MM-014 and 60% of patients in ICARIA-MM received lenalidomide in their most recent regimen prior to study enrollment; nearly all had lenalidomide-refractory disease [58, 59]. The median PFS was 30.8 months in patients treated with daratumumab, pomalidomide, and low-dose dexamethasone in the MM-014 trial [60]. Additionally, all patients in the IC 2013-05 study were in first relapse after lenalidomide-containing induction therapy plus lenalidomide maintenance; however, none had progressed on lenalidomide maintenance, as the duration of maintenance therapy was limited in the Intergroupe Francophone du Myélome (IFM) 2009/Dana Farber Cancer Institute (DFCI) trial [61]. The inclusion of patients who were only exposed, but did not have disease that was refractory, to lenalidomide maintenance after ASCT may account for the outcomes associated with 9 cycles of pomalidomide-cyclophosphamide-dexamethasone followed by pomalidomide-dexamethasone alone (median PFS in arm B, 24.7 months). Prior exposure to daratumumab was a less common consideration due to its recent approvals (1 patient treated with isatuximab-pomalidomide-dexamethasone in ICARIA-MM and isatuximab-carfilzomib-dexamethasone in IKEMA), but patients previously treated with daratumumab were excluded in the MM-014 trial [58].

It is important to acknowledge the potential for downstream sequelae following frontline treatment with lenalidomide. For example, in the CASTOR trial, the median PFS associated with either treatment was lower in patients who developed disease that was refractory to an immunomodulatory agent than in those without refractory disease (median PFS with daratumumab-bortezomib-dexamethasone, 9.2 vs 12.3 months, respectively; median PFS with bortezomib-dexamethasone, 5.4 vs 7.4 months). The treatment duration may also play a role in downstream treatment responses. Patients in the MM-014 trial who received >24 months of prior lenalidomide treatment had a higher 1-year PFS rate compared with patients with ≤24 months of prior lenalidomide treatment (1-year PFS rate, 85.6 vs 64.1%, respectively). The benefits of increased durations of lenalidomide maintenance therapy in prolonging PFS have been seen previously [20, 62]. Compared with the 1-year duration of lenalidomide maintenance therapy in the IFM 2009/DFCI trial, an increased duration of lenalidomide maintenance therapy in the DETERMINATION trial resulted in a greater median PFS (35.0 vs 46.2 months with lenalidomide-bortezomib-dexamethasone, respectively) [62]. Thus, while there are benefits of early-line treatment with lenalidomide, selection of an appropriate regimen following disease progression can be difficult, and the efficacy of these regimens may depend on the duration of prior exposure to immunomodulatory agents. Careful regimen selection following disease progression may help to ameliorate some of these concerns. For example, in the CANDOR trial, patients with prior lenalidomide exposure or lenalidomide-refractory disease had a median PFS of 25.9 or 28.1 months, respectively, with carfilzomib-daratumumab-dexamethasone [63].

Treatment for patients with RRMM varies, as some patients have received prior stem cell transplants, which dictates up-front drug regimen selection, in particular the timing and dosing of lenalidomide. It is not always clear whether individual patients enrolled in clinical trials for RRMM had previously received lenalidomide at full dose as part of induction, or at a lower dose as part of maintenance. Titration of the dose of lenalidomide in maintenance therapy based on toxicity concerns can result in additional variability. In a retrospective analysis, no differences in response or survival rates were reported for patients receiving pomalidomide-dexamethasone after developing disease resistant to different doses of lenalidomide (5–15 vs 25 mg); [64] these results, however, were not directly applicable to lenalidomide maintenance due to the limited number of patients with progression on lenalidomide monotherapy. Among prior studies, 50 to 70% of patients had received prior ASCT, with the MM-014 and ICARIA-MM studies including subgroup analyses based on prior ASCT (Table 2) [58, 59]. In an analysis of the phase 2 EMN011 trial, 95% of the first 60 patients enrolled had progressed on lenalidomide maintenance [55]. In the MM-014 trial, prior dose of lenalidomide was reported; it is difficult to determine, however, if the prior dose was a maintenance or full dose since the starting dose was not published [58]. In the OPTIMISMM trial, the dose of lenalidomide at the time of disease progression was not reported [45]. Consequently, selection criteria for clinical trials have become highly relevant. We believe subsequent trials should focus on patients who have disease refractory to lenalidomide and/or daratumumab at first relapse, as the decision for the next line of therapy is critical. Other factors, such as prior treatments and the lenalidomide dose to which the disease was refractory, are also important considerations. Additionally, the impact on treatment response in RRMM is still being evaluated in patients who received frontline treatment with anti-CD38 agents.

The toxicity profile of these regimens in patients experiencing their first relapse is an additional important consideration, particularly in patients who have undergone ASCT and received only lenalidomide maintenance therapy. Reasons for this include that these patients may have had their relapse detected at a relatively low disease burden due to ongoing monitoring, and these patients usually have control of myeloma-related symptoms and may therefore have been able to resume many pre-diagnosis activities. As such, in the absence of high-risk and/or aggressive disease, the administration schedule and adverse event (AE) profile of the next regimen may be a significant factor in the decision (Table 4). For patients with anticipated responsiveness to an anti-CD38 antibody, the combination of daratumumab or isatuximab with pomalidomide may be an attractive option.

**Table 4:** Toxicity considerations across phase 2 and 3 RRMM clinical trials^a^.

Trial	OPTIMISMM *N* = 559 [45]	MM-014 *N* = 112 [58]	APOLLO *N* = 304 [49]	ENDEAVOR *N* = 929 [41]	CASTOR *N* = 498 [50]	CANDOR *N* = 466 [96]	ICARIA-MM *N* = 307 [59]	IKEMA *N* = 302 [52, 53]
**Drug regimen**	**POM/DEX** + **BORT** ***n*** = **281**	**DARA** + **POM/DEX** ***n*** = **112**	**DARA** + **POM/DEX** ***n*** = **151**	**CFZ** + **DEX** ***n*** = **463**	**DARA** + **BORT/DEX** ***n*** = **251**	**DARA** + **CFZ/DEX** ***n*** = **308**	**ISA** + **POM/DEX** ***n*** = **152**	**ISA** + **CFZ/DEX** ***n*** = **179**
**AEs leading to discontinuation, %**	10.7	3.6	1.9–2.0	29.6	7.4–10	22	7.2	8.4
**Fatal AEs, %**	9.6^b^	1.8	7.3	6.9	5.3	9-10.0	<1–1	3.4

*AE* adverse event, *BORT* bortezomib, *CFZ* carfilzomib, *DARA* daratumumab, *DEX* dexamethasone, *ISA* isatuximab, *POM* pomalidomide, *RRMM* relapsed/refractory multiple myeloma.

^a^Direct comparison between trials is not intended and should not be inferred.

^b^Deaths due to causes other than myeloma during treatment period.

### Future directions for therapy in RRMM

Additional therapies and investigations currently underway or recently approved as new therapeutic options for patients with RRMM include CAR T-cell–based strategies, ADCs, bispecific T-cell engagers, and CELMoD agents. CAR T-cell strategies have demonstrated unprecedented clinical activity in heavily pretreated patients, although a number of practical challenges remain, such as toxicity, time to manufacture, cost, and durability of response [65]. The target of most CAR T-cell trials is BCMA, owing to its higher expression in plasma cells and minimal expression in other tissues [66]. Ide-cel (bb2121) was recently approved by the EC, Health Canada, and FDA in patients after ≥3 [67, 68] or ≥4 [69] prior lines of therapy, including an immunomodulatory agent, a PI, and an anti-CD38 mAb [67–69]. Approvals were based on results of the phase 2 KarMMa trial, which demonstrated an ORR of 73%, a complete response rate of 33%, a median PFS of 8.8 months, and a median OS of 19.4 months at a target dose of 150 to 450 × 10^6^ CAR T cells [70]. In addition to ide-cel, cilta-cel (JNJ-68284528; approved for the treatment of RRMM in the United States, European Union, and Japan) is another BCMA-directed CAR T-cell strategy that has led to impressive response rates and tolerable safety profiles in RRMM, primarily in phase 1 trials [65, 71]. Of note, cilta-cel demonstrated early and deep responses (ORR, 95%; 95% CI, 75–100%; very good partial response or better, 85%; 95% CI, 62–97%) and a manageable safety profile (hematologic AE rate ≥20%: neutropenia [any grade, 95%; grade 3/4, 90%], thrombocytopenia [any grade, 80%; grade 3/4, 35%], anemia [any grade, 65%; grade 3/4, 40%], lymphopenia [any grade, 60%; grade 3/4, 55%], and leukopenia [any grade, 55%; grade 3/4, 55%]) in patients with the lenalidomide-refractory disease who received 1 to 3 prior lines of therapy, including a PI and immunomodulatory drug in cohort A of the multicohort phase 2 CARTITUDE-2 trial [72]. The preliminary safety and efficacy of cilta-cel in CARTITUDE-2 are supportive of the results of CARTITUDE-1, and further investigation is ongoing in phase 3 CARTITUDE-4 trial [71, 72]. Additionally, with the success of daratumumab, CAR-CD38 T cells are being studied in preclinical trials [12], and a phase 1 study of BCMA-CD38 dual-target CAR T cells reported an ORR of 83% [73]. CAR T cells targeting GPRC5D have also shown preliminary efficacy in the MCARH109 phase 1 study in patients with RRMM who had received ≥3 lines of treatment [74].

ADCs and bispecific T-cell engagers targeting BCMA are also novel treatment approaches in MM [1]. BCMA-targeted ADCs include belantamab mafodotin-blmf and CC-99712. Belantamab mafodotin-blmf had received accelerated FDA approval in patients with RRMM; however, the application for market approval was recently withdrawn based on the results of the DREAMM-3 trial [75, 76]. CC-99712 evaluation is underway in a phase 1 first-in-human trial in patients with RRMM who received ≥3 prior therapies [77].

Bispecific T-cell engagers or bispecific mAbs targeting BCMA currently being investigated in phase 1 clinical trials in later lines of therapy include the following (with associated median [range] of prior lines of therapy): AMG420 (7 [3–14]), [78], AMG701 (6 [1–25]), [79], CC-93269 (5 [3–13]), [80], teclistamab (6 [2–14]), [81], REGN5458 (not available (NA) [3-NA]), [82], elranatamab (8 [NA]), [83], and TNB-383B (7 [4–13]. [84]. In a phase 1/2 study of teclistamab, patients with RRMM who had received ≥3 prior lines of treatment achieved a median PFS of 11.3 months[81]. Teclistamab has been approved in Europe as a monotherapy for patients with RRMM who have received ≥3 prior lines of therapy and in the United States for patients with RRMM who have received ≥4 prior lines of therapy, including a proteasome inhibitor, an immunomodulatory agent, and an anti-CD38 mAb. Bispecific T-cell engagers targeting GPRC5D, an orphan G protein–coupled receptor highly expressed in MM cells, (talquetamab) [85], and Fc receptor-homolog 5 (FcRH5), a type I membrane protein expressed on B cells, plasma cells, and nearly all MM cells, (cevostamab [BFCR4350A]) [86] are also being evaluated in phase 1 trials in MM in later lines of therapy.

CELMoD agents target the same pathway as lenalidomide and pomalidomide but are more potent and act more rapidly. The CELMoD agents iberdomide and mezigdomide have shown activity in MM cell lines that are resistant to lenalidomide and pomalidomide [87, 88]. A phase 1b/2a study of iberdomide-dexamethasone demonstrated preliminary efficacy and safety in patients who received a median of 5 (range, 2–12) prior lines of therapy [89]. Additionally, a phase 1 study of mezigdomide-dexamethasone reported preliminary activity in patients who received a median of 6 (range, 2–13) prior lines of therapy, including lenalidomide (97%) [90].

Drugs with other novel MOAs in RRMM include selinexor and venetoclax [18, 19, 91]. Selinexor, after having been granted prior accelerated approval in combination with dexamethasone in patients who received ≥4 prior therapies with disease refractory to ≥2 immunomodulatory agents, ≥2 proteasome inhibitors, and an anti‐CD38 mAb, was recently approved in combination with bortezomib-dexamethasone in patients who received ≥1 prior therapy [19]. This approval was based on the phase 3 BOSTON trial, which demonstrated a 13.9-month median PFS in patients who received a median of 2 (range, 1–3) prior lines of therapy [43]. Venetoclax is not FDA approved for MM but has demonstrated improvement in PFS in combination with bortezomib-dexamethasone in patients with RRMM who received 1 to 3 prior therapies in the phase 3 BELLINI trial, with subgroup analyses suggesting promising activity in patients with translocation t(11;14) [91].

Another promising candidate treatment for patients with RRMM is modakafusp alfa (TAK-573), a first-in-class “immunocytokine” designed to deliver interferon alpha-2b (IFNα2b) to CD38^+^ cells. This agent, consisting of 2 (attenuated) IFNα2b molecules fused to the Fc portion of a humanized, anti-CD38 mAb, is designed to induce direct antiproliferative effects on myeloma cells and cause both direct and indirect immune cell activation [92]. A first-in-human phase 1 trial of modakafusp alfa monotherapy showed promising efficacy in 59 patients with RRMM; of the 24 patients treated with 1.5 mg/kg modakafusp alfa once every 4 weeks, neutropenia (50%), leukopenia (38%), decreased lymphocyte count (38%), anemia (33%), and thrombocytopenia (33%) were the most frequent grade 3/4 treatment-emergent AEs.

## Conclusion

With the treatment landscape in MM rapidly changing in response to approvals of new drugs and additional combinations, it is important to focus on patients with disease refractory to early-line lenalidomide and/or daratumumab, the most common population in clinical practice today. Variations in patient baseline characteristics in recent clinical trials, such as the number of prior lines of treatment, refractoriness to prior treatment, and dose of prior lenalidomide, make it difficult for clinicians to choose the best options for their patients with RRMM for whom first-line treatments have failed.

**Fig. 1:**
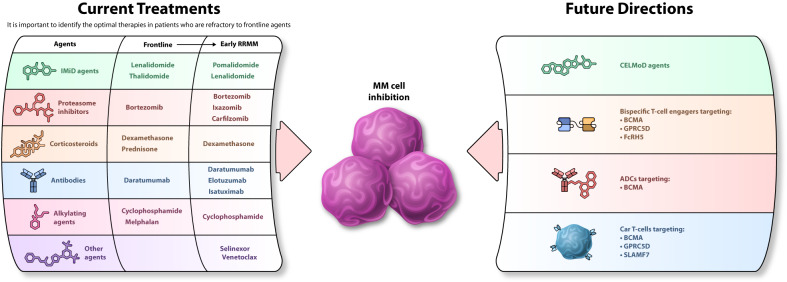
Overview of current treatment options and future directions in the early RRMM treatment landscape. The agents commonly used for treatment of patients with multiple myeloma in frontline and early relapsed/refractory settings are presented on the left (Current Treatments); agents being evaluated in clinical trials or entering the treatment space are described on the right (Future Directions). ADC antibody-drug conjugate, BCMA B-cell maturation antigen, CAR chimeric antigen receptor, CELMoD cereblon E3 ligase modulating drug, FcRH5 Fc receptor-homolog 5, GPRC5D G-protein coupled receptor family C group 5, mAb monoclonal antibody, MM multiple myeloma, RRMM relapsed/refractory multiple myeloma, SLAMF7 SLAM family member 7.

Currently, pomalidomide-, carfilzomib-, and anti-CD38–based regimens are good options for patients with RRMM; [2, 6, 7, 9] however, more focus is needed specifically on patients previously exposed or refractory to early-line lenalidomide and/or daratumumab. Treatment combination options currently being considered for these patients include bortezomib or carfilzomib added to pomalidomide-dexamethasone. In addition, for patients with the lenalidomide-refractory disease who have also been exposed (although not refractory) to a fixed duration of daratumumab until progression, the addition of daratumumab or isatuximab to either pomalidomide-dexamethasone or carfilzomib-dexamethasone may be considered. Finally, for patients with the daratumumab-refractory disease who were also exposed, but not refractory, to lenalidomide, potential options include carfilzomib, isatuximab, or elotuzumab added to pomalidomide-dexamethasone or lenalidomide-dexamethasone. After exposure to immunomodulatory agents, PIs, and anti-CD38 mAbs, and refractoriness to lenalidomide and/or daratumumab, BCMA-targeted therapy (CAR T cells or T-cell engagers) may be considered (Fig. 1) [69, 70]. In addition, the timing of daratumumab administration within quadruplet regimens is being investigated to determine whether frontline inclusion has a more beneficial survival benefit vs at relapse [25]. Consequently, the clinical trial design will need to be tailored to adapt to the evolving treatment landscape.

Within the next few years, it is expected that CAR T cells targeting BCMA, GPRC5D, and SLAMF7 will also enter the RRMM and NDMM treatment landscapes, especially with the approval of ide-cel in the United States, European Union, Canada, and Japan and the approval of cilta-cel in the United States, Japan, and Europe [67–69, 93]. CAR T-cell therapy, however, has several limitations, such as availability, manufacturing time, the need for bridging therapy, good performance status and satisfactory organ function requirements, cost of therapy, and the need for family and social support. CAR T cells are also moving into earlier lines of therapy and focusing on high-risk patient populations, with maintenance approaches after CAR T-cell therapy under investigation. Bispecific T-cell engagers are also off-the-shelf products with broad availability and are a good first choice for patients with aggressive disease who are unable to wait for CAR T-cells. Although good performance status and adequate organ function are required for these, they can be administered to patients with comorbidities or disabilities that may have precluded the use of CAR T-cell therapy. Additionally, the use of bispecific T-cell engagers requires hospitalization for the priming doses and the first full dose to manage toxicity, but administration at a specialized center is not required for subsequent doses. Another consideration relates to whether patients will be limited to only 1 BCMA-targeted therapy due to efficacy concerns or eligibility requirements. If this is the case, the advantages and toxicities of different immunotherapy platforms will become important determinants in treatment selection. Complementary strategies that may be considered for treatment for early RRMM in the future include bispecific T-cell engagers combined with CAR T cells, immunomodulatory agents such as CELMoD agents, or other mAbs.

## Data Availability

Data sharing is not applicable as no relevant datasets were generated for the development of this article.
