# Correction: Clinical evidence for immune-based strategies in early-line multiple myeloma: current challenges in decision-making for subsequent therapy

**DOI:** 10.1038/s41408-023-00866-y

**Published:** 2023-07-06

**Authors:** Noopur Raje, María-Victoria Mateos, Shinsuke Iida, Donna Reece

**Affiliations:** 1 Department of Medical Oncology, Massachusetts General Hospital, Boston, MA USA; 2 University Hospital of Salamanca/IBSAL/Cancer Research Center-IBMCC (USAL-CSIC), Salamanca, Spain; 3 Department of Hematology and Oncology, Nagoya City University Graduate School of Medical Sciences, Nagoya, Japan; 4 Princess Margaret Cancer Centre, Toronto, ON Canada

**Keywords:** Haematological diseases, Myeloma

Correction to: *Blood Cancer Journal* 10.1038/s41408-023-00804-y, published online 22 March 2023

In the sentence beginning ‘Patients in the MM-014 trial who received...’ in this article, the text ‘lower’ should have read ‘higher’.

Moreover, the sentence beginning ‘However, these considerations should be balanced’ should have been replaced with the following sentence.

“The benefits of increased durations of lenalidomide maintenance therapy in prolonging PFS have been seen previously.”

The original article has been corrected.

